# Health system resilience and pandemic response: a comparative analysis of China, Singapore, the U.S., and the U.K.

**DOI:** 10.3389/fpubh.2025.1666323

**Published:** 2025-09-01

**Authors:** Shupeng Lyu, Chen Qian, Ling Yuan, Zhidong Yuan, Ching-Hung Lee

**Affiliations:** School of Public Policy and Administration, Xi'an Jiaotong University, Xi'an, China

**Keywords:** COVID-19, resilience, resilience from scale, resilience from structure, health policy

## Abstract

**Introduction:**

Infectious disease outbreaks have imprinted unprecedentedly on global economies, societies, politics, and healthcare systems. The COVID-19 pandemic underscored critical challenges in global healthcare delivery, necessitating the translation of lessons into actionable strategies for strengthening health system resilience against future outbreaks.

**Methods:**

This paper divides resilience into two dimensions: resilience from scale and resilience from structure. The former pertains to the overall resilience of the “state-society” system, while the latter refers to resilience rooted in the system's internal structure. Expert consultation method is used to assess the potential and actual levels of two types of resilience. The case study and time slicing approach are used to analyze the anti-epidemic policies in four countries.

**Results:**

There are significant differences in the potential and actual levels of resilience from scale and resilience from structure in the event of infectious disease outbreaks in China, Singapore, the U.S., and the U.K., as a result of a combination of political and non-political factors. Based on the original perspective of two types of resilience, this study reveals that differences in anti-epidemic policies among these countries stem from variations in the resilience from scale and resilience from structure.

**Conclusion:**

This paper elucidates the divergent global responses to the same virus from the original perspective of two types of resilience. Furthermore, the study presents a practice-oriented framework that links health system scale and structure to anti-epidemic policies, thereby moving beyond existing indices like the Global Health Security Index. The findings deliver concrete lessons for improving managerial practices, enhancing preparedness, and informing future healthcare delivery innovations, directly contributing to translating pandemic experience into implementable best practices for strengthening health systems against infectious disease threats.

## 1 Introduction

In October 2019, the Johns Hopkins Center for Health Security, the Nuclear Threat Initiative, and the Economist Intelligence Unit jointly released the Global Health Security Index (hereafter GHS Index). It comprehensively assessed the capacity of 195 countries to prevent and mitigate epidemics and pandemics. According to the index, the United States (hereafter the U.S.) and the United Kingdom (hereafter the U.K.) ranked first and second with a score of 83.5 and 77.9, respectively. Singapore ranked 24th with a score of 58.7. China ranked 51st with a score of 48.2. In a highly dramatic turn of events, a few months after the release of the index, the coronavirus disease 2019, i.e., COVID-19 spread rapidly across the globe causing serious concerns. On March 11, 2020, the World Health Organization (hereafter WHO) declared COVID-19 a global pandemic. COVID-19 prevention and control might be regarded as a test of the governance system and governance capacity of the countries in the world, as well as a review of the GHS Index.

The policies for preventing and controlling COVID-19 varied greatly across the globe due to differences in political systems, economic systems, and cultural values (1, 2). Comparative analysis reveals distinct national approaches. For instance, China, the largest authoritarian country in the contemporary world, is a representative of one adopting the “zeroing” policy (3–5). The implementation of China's “dynamic zeroing” policy was followed by periods of lower infection rates, fatality rates, and excess deaths. However, this approach also incurred significant economic, social, and human rights costs. At the end of 2022, after gaining a window of opportunity, i.e., the waning virulence of the Omicron variant, improved treatment capabilities, and mass vaccination, China officially ended its “dynamic zeroing” policy and lifted all NPIs, formally entering the stage of coexistence with COVID-19. On the contrary, some of the 13 countries in the GHS Index that were most prepared for epidemics and pandemics, restricted by their own political systems, economic systems, and cultural values, had already chosen to coexist with the virus in the early stages of the outbreak when its fatality rate was still high (6, 7). Their fight against COVID-19 was focused on slowing the spread of the virus as much as possible and flattening the curve of new infections, aiming at reducing the strain on healthcare resources and preventing healthcare systems from being overwhelmed (8). This could be understood in essence as a return to social Darwinism (9, 10). However, until effective therapeutics and vaccines have been developed and the virus has mutated to become more benign, this strategy has led to the widespread spread of the virus. Documented outcomes include large numbers of deaths and excess deaths, alongside worsened existing inequalities in terms of income, health, safety, and more (11, 12).

On May 5, 2023, the WHO announced that COVID-19 no longer constituted a public health emergency of international concern (PHEIC). Currently, most countries in the world have ended the interventions that have been used to contain COVID-19 on a societal level. While this does not mean the complete end of COVID-19 as a global health threat, it proves that the impact of this disease on human life and healthcare systems has been reduced to a tolerable level. However, it is highly likely that COVID-19 will not be the last infectious disease pandemic (13). Therefore, it is imperative to ensure that countries do not give up on learning the lessons of COVID-19 prevention and control and to strengthen their preparedness for future epidemics and pandemics. This imperative raises crucial questions central to enhancing healthcare delivery during crises: why did different countries adopt quite different policies? Why is there such a difference in the capacity of countries to prevent and mitigate epidemics and pandemics between the theoretical level interpreted by the GHS Index and the realistic level in response to COVID-19? Against the backdrop of the accelerated evolution of the world's major changes unseen in a century and the increasingly intense great power games, understanding these differences is vital not only for global health governance but, more critically, for informing concrete managerial innovations and health system redesign to strengthen future epidemic response quality (14, 15). To address these questions, this study selects China, Singapore, the U.S., and the U.K. as case studies and analyzes the differences in the anti-epidemic policies of these countries and attempts to answer the above questions based on the original perspective of “two types of resilience.”

The paper is organized as follows. After an introduction of the materials and methods in Section 2, the results are elaborated in Section 3, and discussion and conclusions are provided in Section 4.

## 2 Materials and methods

### 2.1 Research framework

The concept of resilience was introduced into the field of risk and disaster management at the end of the last century and was described as the ability of an organization or system to withstand the effects of a disaster and recover (16). To better analyze the logic behind the differentiated prevention and control strategies of different countries, this paper further divides resilience into two dimensions: resilience from scale and resilience from structure. Resilience from scale and resilience from structure constitute the two core dimensions of system resilience, which are intrinsically distinct from and functionally influenced by each other. They collectively shape the ability of the coupled “state-society” system to withstand, resist, and recover from infectious disease outbreaks. The coupled “state-society” system refers to an organic whole formed by the close connections and mutual interactions between state institutions and social forces, which is characterized by their structural and functional interweaving at a deep level.

**Figure 1:**
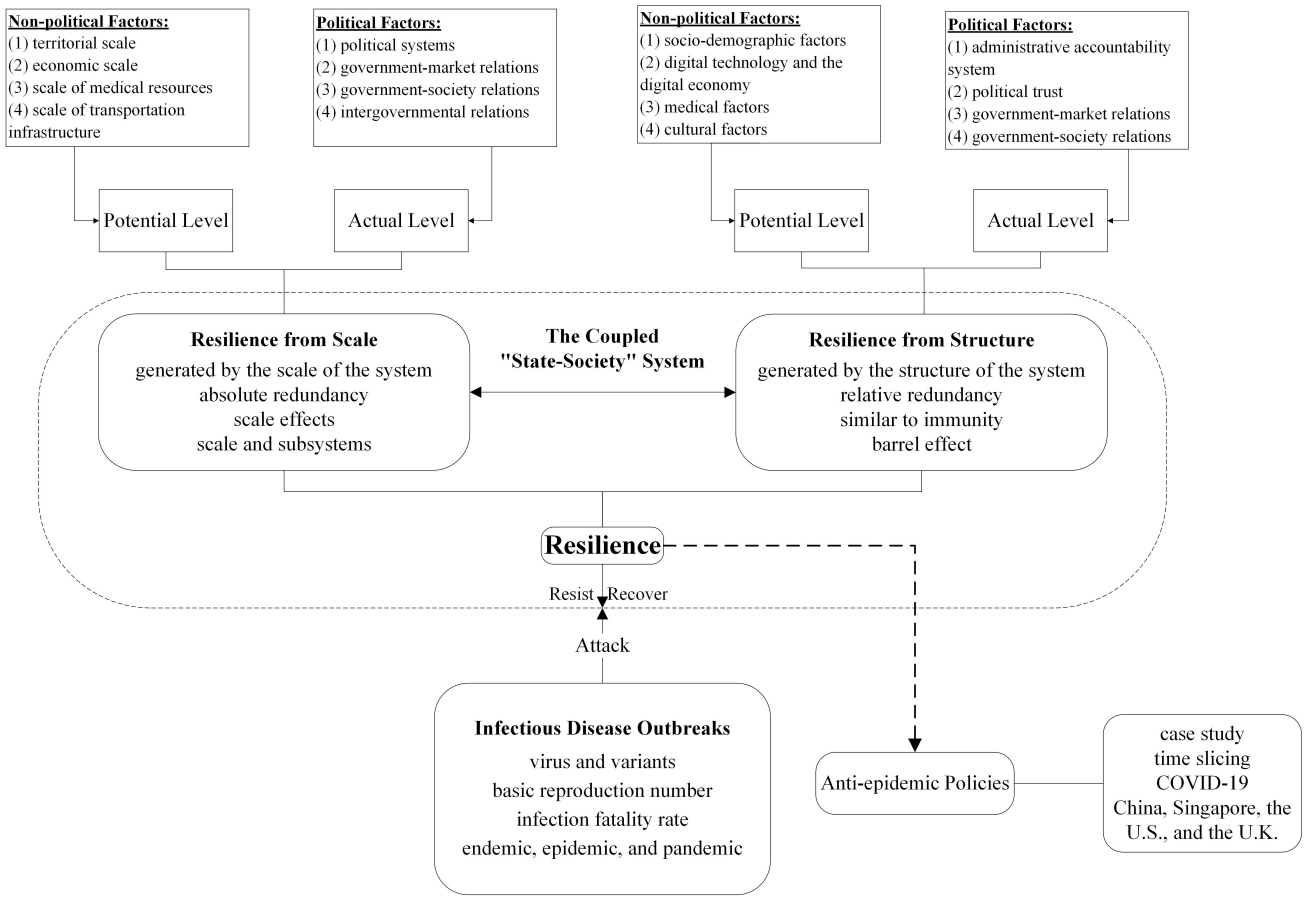
Research framework.

The research framework of this paper is shown in Figure 1. Based on the definition of the two types of resilience, this paper proposes an evaluation scheme for assessing the potential and actual levels of the two types of resilience in the case of infectious disease outbreaks, integrating both political and non-political dimensions. Specifically, guided by the principles of data availability, comparability, and systematicity, this paper delineates key influencing factors across the political and non-political dimensions. Potential levels for each resilience type are derived from quantifiable, spatiotemporally comparative non-political factors. Non-political factors affecting resilience from scale include territorial scale, economic scale, medical resource scale, and transportation infrastructure scale. Meanwhile, non-political factors affecting resilience from structure include the socio-demographic characteristic, digital technology, medical characteristic, and cultural characteristic. Each factor can independently influence either resilience type. Potential levels of two types of resilience are then determined by aggregating all corresponding factor assessments. Subsequently, these potential levels are modified by non-quantifiable political factors, yielding context-sensitive actual levels. Political factors affecting resilience from scale include political system, government-market relations, government-society relations, and intergovernmental relations. Meanwhile, political factors affecting resilience from structure include the administrative accountability system, political trust, government-market relations, and government-society relations. Similarly, each factor exerts an independent effect on either resilience type. Actual levels of two types of resilience are then established through systematic aggregation of all relevant factor assessments. Building upon this foundation, this study undertakes a systematic examination of variations and nuances in the two types of resilience as well as public health policies implemented to combat the COVID-19 pandemic across four representative countries. Through a phased analysis, this study dissects pandemic response trajectories across four countries to systematically investigate how underlying differences in the two types of resilience fundamentally shape divergent policy pathways, particularly in terms of rigidity-flexibility tradeoffs, innovation adoption speed, and crisis recalibration efficiency.

### 2.2 Case selection and methodology

This study employs the comparative case study approach to investigate the manifestations of two types of resilience as well as anti-epidemic strategies across four countries: China, Singapore, the U.S., and the U.K. The selection of these four countries is primarily based on the following considerations. First, they exhibit pronounced variations in the influencing factors of the two types of resilience, especially the political system and culture. These significant differences enable us to transcend the limitations of single-case studies and effectively capture and compare the distinct manifestations of the two types of resilience across diverse contexts. Second, significant divergences exist in the evolution trajectories and implementation outcomes of anti-epidemic policies across these four countries. Selecting these countries as research subjects not only satisfies the core requirements of representativeness and heterogeneity essential for case studies but also provides a critical reference for formulating context-specific response strategies in future global public health crisis management. Third, these four countries possess relatively abundant, accessible, and reliable data during the pandemic period, including cases, deaths, social survey results, and official policy documents, which provides the necessary foundation for rigorous analysis.

To assess the two types of resilience, this study employs the expert consultation method. Expert selection adheres to three criteria to ensure representativeness and expertise: (1) Sustained research activity or extensive practical experience in infectious disease prevention and control, emergency management, or healthcare innovation; (2) Current employment at core organizations, including hospitals, emergency management agencies, and academic research institutions; (3) Primary location in the Northwest China, with representation also from other areas. Based on these criteria, seven experts are ultimately selected to participate in the consultation. The Delphi method is used for the consultation process. All experts are provided with comprehensive background materials, including the research context and objectives, theoretical framework, specific indicator system, and preliminary assessment results for two types of resilience. Two rounds of anonymous consultations are then conducted. The first round employs an open-ended format, prompting experts to independently critique both the initial assessments (based solely on the non-political dimension) and the subsequent revisions (after incorporating the political dimension) for two types of resilience. Experts then provide their specific modification suggestions. The second round focuses on key points of disagreement and revision proposals identified in the first round, guiding experts toward consensus or clarification of divergent viewpoints. Ultimately, the consensus and key modifications derived from this process serve to recalibrate the assessment of both potential and actual levels of two types of resilience.

In parallel, the time-slicing method is utilized to map and analyze the evolution of anti-epidemic strategies in the four countries. It segments a continuous time series into homogeneous, non-overlapping stages based on specific criteria, thereby facilitating understanding of differences and evolutionary trends between stages. In this study, the stage division of the practice in COVID-19 prevention and control across the four countries is primarily based on epidemiological characteristics (e.g., changes in the dominant strain) and critical policy shifts (e.g., nationwide lockdown/reopening). This paper follows three principles in defining temporal stages. First, specific time points and stage divisions vary according to the actual circumstances of each country. Second, anti-epidemic policies should remain predominantly stable and consistent throughout each defined stage. Third, these divisions should be aligned with the research purposes of this study, including analyzing resilience performance and policy evolution. Specifically, this study systematically compiles key events and policy measures implemented by four countries in the practice of preventing and controlling COVID-19. Drawing on distinct epidemiological characteristics and critical policy shifts, and referencing key data, including the Oxford COVID-19 Government Response Tracker, alongside WHO-published figures on confirmed cases, deaths, excess deaths, and vaccination rates, this paper defines distinct anti-epidemic stages for each country. We then accurately allocate events and policy measures to their corresponding stages based on chronological order. Subsequently, we identify the defining characteristics of each stage. On this basis, the study analyzes temporal variations in key outcome indicators across the different stages to evaluate the efficacy of the implemented policies. By identifying key policy inflection points and their corresponding outcomes, this paper aims to facilitate a dynamic understanding of how resilience shapes policy response and implementation.

### 2.3 Definitions and factors influencing two types of resilience

#### 2.3.1 Resilience from scale

In this paper, resilience from scale is defined as the ability of a coupled “state-society” system to resist, respond to, and recover from the impacts of infectious disease outbreaks as a result of the elements of scale, such as territorial area, population size, and economic volume. Differing from the general concept of resilience, resilience from scale focuses on revealing how scale-dependent properties within coupled “state-society” systems function as key drivers for the generation, manifestation, and evolution of resilience. This framework posits scale not as a mere background parameter describing system attributes, but as an interpretive lens shaping system resilience in infectious disease outbreak settings.

The underlying logic of resilience from scale lies in the fact that disasters have a certain scale, which is manifested in a specific scope of destruction and impact. Meanwhile, the objects targeted by disasters also have a certain scale. When a small-scale disaster occurs in a large-scale country, even if this disaster causes significant damage to localized areas, these affected areas can quickly draw support from other areas, resulting in a strong coping and recovery capacity, i.e., a high level of resilience from scale. Conversely, when a large-scale disaster occurs in a small-scale country, the impact of the disaster may cover most, if not all, of the country, resulting in a weak coping and recovery capacity due to the scarcity of its own resources coupled with the inability to obtain support from other areas of the country, i.e., a low level of resilience from scale. In addition, the definition of resilience from scale dictates an important feature, namely that the resilience from scale of each subsystem embedded in the system can be significantly enhanced as the scale of the system increases. For instance, the 2008 Wenchuan earthquake caused 69,227 deaths, 17,923 missing, 374,643 injuries, and more than 845 billion yuan in direct losses, making it the most destructive in China since the 1950s. Sichuan Province demonstrated a high level of resilience from scale with the support from the central government and other provinces. Within 3 years, Sichuan Province rebuilt housing for more than 5.4 million affected families, reconstructed 2,989 schools, and built 1,359 new healthcare and rehabilitation facilities. In contrast, after earthquakes of similar magnitude and comparable destructive power struck small nations such as Nepal (magnitude 8.1 in 2015) and Haiti (magnitude 7.3 in 2021), their affected areas experienced significantly longer recovery times for restoring economies and infrastructure to pre-earthquake levels. This protracted recovery stemmed primarily from constrained resilience from scale, where limited fiscal resources and logistical bottlenecks hindered the mobilization of timely and adequate domestic assistance.

The non-political factors affecting resilience from scale can be summarized as the following four aspects. The first factor is territorial scale. The larger the territorial scale, the less impact on the whole country the implementation of social control measures such as lockdowns and traffic controls in an infected area will have, and the easier it will be to gain the support of public opinion. The second factor is economic scale. The larger the economic scale, the greater the ability of the country to withstand disasters of a given scale on its own, rather than always counting on international aid. The third factor is the scale of medical resources. Countries with abundant medical resources are not only able to form a more specialized team of medical researchers to promote the development of vaccines and antiviral drugs but also to isolate and treat infected cases to the greatest extent possible. The fourth factor is the scale of transportation infrastructure. The larger the scale of the transportation infrastructure, the more conducive it is for the country to deploy supplies and medical personnel from various areas during emergencies.

The political factors affecting resilience from scale can be summarized as the following four aspects. The first factor is the political system. The political system plays an important role in organizing and mobilizing forces, developing and implementing NPIs, and allocating scarce resources (17). The second factor is government-market relations. It can influence resilience from scale because the response to an outbreak requires the efficient pooling and deployment of advantageous resources within the market and integration of these into a force of significant scale to combat the outbreak. The third factor is government-society relations. In the case of infectious disease outbreaks, government-society relations play a critical role in the effective dissemination of directives and information, efficient implementation of anti-epidemic policies, and extensive mobilization of social forces. The fourth factor is intergovernmental relations. Good inter-governmental cooperation and mutual assistance can help to improve outbreak response capacity (18).

#### 2.3.2 Resilience from structure

In this paper, resilience from structure is defined as the ability of a coupled “state-society” system to resist, respond to, and recover from the impacts of infectious disease outbreaks as a result of the elements of structure, such as institutions and mechanisms, laws and regulations, and medical technology. Differing from the general concept of resilience, resilience from structure focuses on revealing how structure-dependent properties within coupled “state-society” systems function as key drivers for the generation, manifestation, and evolution of resilience. This framework posits structure not merely as a reflection of a system's fundamental framework and operational logic, but rather as the shaping mechanism that enables the system's resilience traits in infectious disease outbreak settings.

The underlying logic lies in the fact that disasters have a certain degree of intensity and destructive power, while their target objects rely on their own internal structure to produce a certain degree of resistance and recovery, so as to be able to effectively withstand disasters or to recover quickly after suffering a disaster. When the intensity or destructive power of a disaster is less than a certain threshold, the damage it causes is limited and manageable, and the system can still remain relatively stable. When the intensity or destructive power of a disaster exceeds a certain threshold, the system with a low level of resilience from structure may face the risk of complete collapse, while the system with a high level of resilience from structure is able to realize self-repair and return to the state of orderly operation. In addition, the definition of resilience from structure dictates an important feature, namely that the level of resilience from structure is limited by the weakest link in the system's structure. This phenomenon is commonly known as the “buckets effect,” illustrating that the water capacity of a wooden bucket composed of multiple staves is determined by the shortest stave rather than the longest. The 2010 Mw 8.8 Chile earthquake and tsunami served as a striking example. Significant deficiencies in tsunami defenses were revealed, particularly in the critical industrial port of Talcahuano. Talcahuano's structural protections were likely insufficient for a maximum credible tsunami, while flaws in warning dissemination and response protocols were also apparent. Consequently, catastrophic damage to this infrastructure triggered severe cascading effects, significantly impeding Chile's nationwide recovery efforts.

The non-political factors affecting resilience from structure can be summarized as the following four aspects. The first factor is the socio-demographic characteristic. Other things being equal, countries with moderate population densities, lower proportions of vulnerable populations, higher levels of education, and lower levels of residential agglomeration tend to have higher levels of resilience from structure. The second factor is digital technology. When infectious disease outbreaks occur, countries with advanced and widespread use of digital technologies, a mature contactless economy (e.g., online shopping), and few legal barriers tend to have high levels of resilience from structure. The third factor is the medical characteristic. Other things being equal, countries with more advanced medical technology, higher community health awareness, higher levels of public immunity, and greater per capita medical resources tend to have higher levels of resilience from structure. The fourth factor is the cultural characteristic. This affects the resilience from structure of the system because it is internalized by the majority of the country's population, including the members of the government that formulate policies (19).

The political factors affecting resilience from structure can be summarized as the following four aspects. The first factor is the administrative accountability system. Precise and effective accountability can enhance the effectiveness and efficiency of the fight against the epidemic. The second factor is political trust. The people's high level of trust in the government at all levels means both stronger confidence in the government's ability to control the epidemic, as well as the individual's voluntary obedience to the government's control (20, 21). The third factor is government-market relations. Compared with free-market capitalism, planned economy and state capitalism are more willing to compromise on profits (22). The fourth factor is government-society relations. Government-society relations determine the speed and efficiency of anti-epidemic response, serving as a critical foundation for the prevention and control of infectious disease outbreaks.

## 3 Results

### 3.1 Differences in two types of resilience between four countries

#### 3.1.1 Differences in resilience from scale

Non-political factors are analyzed initially. This study only compares the factors that can be directly quantified, and some of the factors that are difficult to quantify are measured by indicators with higher correlation. This principle applies equally to resilience from structure. First, the territorial areas of China, Singapore, the U.S., and the U.K. are 9.38 million km^2^, 716 km^2^, 9.14 million km^2^, 0.24 million km^2^, and, respectively (23). Meanwhile, from 2019 to 2021, the total population of China was 1,408 million, 1,411 million, and 1,412 million, respectively, that of Singapore was 5.70 million, 5.69 million, and 5.45 million, respectively, that of the U.S. was 328 million, 332 million, and 332 million, respectively, and that of the U.K. was 66.84 million, 67.08 million, and 67.03 million, respectively (23). Second, from 2019 to 2021, the Gross Domestic Product (GDP, current US$) of China was 14.28 trillion, 14.69 trillion, and 17.82 trillion, respectively, that of Singapore was 0.3768 trillion, 0.3484 trillion, and 0.4238 trillion, respectively, that of the U.S. was 21.38 trillion, 21.06 trillion, and 23.31 trillion, respectively, and that of the U.K. was 2.857 trillion, 2.705 trillion, and 3.122 trillion, respectively (23). Third, from 2019 to 2021, the total number of doctors in China was 3.182 million, 3.372 million, and 3.558 million, respectively, that of Singapore was 14.3 thousand, 14.8 thousand, 15.3 thousand, respectively, that of the U.S. was 865.9 thousand, 873.2 thousand, 886.4 thousand, respectively, and that of the U.K. was 197.2 thousand, 203.3 thousand, 213.2 thousand, respectively. From 2019 to 2021, the total number of nurses in China was 4.407 million, 4.656 million, and 4.970 million, respectively, that of Singapore was 42.8 thousand, 42.1 thousand, and 43.1 thousand, respectively, that of the U.S. was 3.926 million, 3.927 million, 3.977 million, respectively, and that of the U.K. was 548.1 thousand, 567.5 thousand, 581.8 thousand, respectively. From 2019 to 2021, the total number of hospital beds in China was 6.8 million, 7.05 million, and 7.34 million, respectively, that of Singapore was 14 thousand, 14 thousand, and 13.5 thousand, respectively, that of the U.S. was 918.4 thousand, 922.9 thousand, 919.6 thousand, respectively, and that of the U.K. was 163.8 thousand, 163.0 thousand, 162.2 thousand, respectively (24). Finally, from 2019 to 2021, the total route of rail lines in China was 102,462, 106,235, and 109,767 km, respectively, that of the U.S. was 149,488, 148,749, and 148,553 km, respectively, and that of the U.K. was 16,295, 16,351, and 16,178 km, respectively (23).

Based on the differences in the objective indicators mentioned above, the authors engage in a thorough discussion and careful analysis. Initial results are derived for the levels of resilience from scale on the four sub-dimensions and the composite dimension. Subsequently, we allow seven experts to conduct the evaluation, examination, and revision of the initial results. Table 1 presents the potential levels of resilience from scale for the four countries under various non-political factors and their combined effects. According to Table 1, the potential levels of resilience from scale in China, Singapore, the U.S., and the U.K. are high, low, high, and medium, respectively.

**Table 1:** Potential levels of resilience from scale.

**Country**	**Territorial scale-derived resilience**	**Economic scale-derived resilience**	**Medical scale-derived resilience**	**Infrastructural scale-derived resilience**	**Potential level under combined effects**
China	High	Moderately high	High	Moderately high	High
Singapore	Low	Low	Low	Low	Low
The U.S.	Moderately high	High	Moderately high	High	High
The U.K.	Medium	Medium	Medium	Medium	Medium

While comparisons based on objective indicators of non-political factors can reduce the difficulty of analysis and increase the neutrality of the results, they are prone to pitfalls. As with the GHS Index mentioned at the beginning of this paper, it may be difficult to assess the actual level of resilience from scale if political factors are ignored. Indeed, the fact that political factors are difficult to quantify does not mean that they cannot be analyzed and compared. Even in terms of actual anti-COVID-19 practices, political factors play a crucial role. Therefore, in order to measure the level of resilience from scale more precisely, this paper modifies the results derived from non-political factors by analyzing the political factors in the four countries.

First, China is an authoritarian country with a unitary political system. The CPC is the only political party in power. Singapore is a unitary parliamentary democracy with the People's Action Party (PAP) in power for a long time. The U.S. is a federal republic with the Democrats and the Republicans alternating in power. The U.K., the representative of the middle countries in Paul Kennedy's book, is a unitary parliamentary democracy and constitutional monarchy. The Labor Party and the Conservative Party alternate in power. Second, China operates as a socialist market economy. The capacity of the government to intervene in the economy is strong. Singapore is a liberal market orientated economy with the strategy of state capitalism. From the outset, the PAP government has been characterized by a high degree of interventionism (25, 26). The U.S. and the U.K. operate as a mixed economy featuring the combination of free markets and a certain degree of economic intervention by the government (27, 28). Third, the governance models of China and Singapore typically exhibit a more pronounced state-directed orientation, emphasizing the central role of the government in policy formulation and societal guidance (29). In contrast, the governance models of the U.S. and the U.K. place greater emphasis on broad participation by diverse societal forces and institutionalized checks on governmental power (30). Finally, there is a strict hierarchical relationship between the central government and local governments in China. State and local governments in the U.S. have a high degree of autonomy in public health governance. Local governments in the U.K. have a greater degree of independence and are responsible for a range of community services, including health and sanitation. Singapore is too small for the discussion of intergovernmental relations.

The authors arrive at an initial revision by discussing and analyzing the changes in the level of resilience from scale in the four countries that may result from political factors. Subsequently, we allow seven experts to conduct the evaluation and modification of the initial revision. Table 2 presents the impact of key political factors on the actual levels of resilience from scale in the four countries during infectious disease outbreaks, revealing how these factors account for deviations between actual levels of resilience from scale and their potential levels. After considering the influence of political factors, we posit that the actual levels of resilience from scale in China and Singapore are higher than their potential levels, while the actual levels of resilience from scale in the U.S. and the U.K. are lower than their potential levels.

**Table 2:** Actual levels of resilience from scale.

**Country**	**Impact of political systems**	**Impact of government-market relations**	**Impact of government-society relations**	**Impact of intergovernmental relations**	**Actual level of resilience**
China	In the event of infectious disease outbreaks, compared with the U.S. and the U.K., the political systems in China and Singapore are conducive to mobilizing the enthusiasm of all parties and ensuring a coordinated national response.	In the event of infectious disease outbreaks, compared with the U.S. and the U.K., China and Singapore find it easier to increase the production and supply of anti-epidemic materials and suspend the private production of wealth.	In the event of infectious disease outbreaks, compared with the U.S. and the U.K., China and Singapore have greater social control, which enables them to implement social control measures, such as lockdowns and stay-at-home orders, more rapidly and rigorously.	In the event of infectious disease outbreaks, compared with the U.S. and the U.K., China and Singapore can enable all governments to implement a unified anti-epidemic strategy and take coordinated and rapid action.	Higher than potential
Singapore					Higher than potential
The U.S.					Lower than potential
The U.K.					Lower than potential

#### 3.1.2 Differences in resilience from structure

Non-political factors are analyzed initially. First, from 2019 to 2021, the population density of China was 149, 150, and 150 people/km^2^, respectively. Meanwhile, according to the Seventh National Population Census in 2020, the population density in the southeast of the Hu Line was 321.87 people/km^2^ (31). From 2019 to 2021, the population density of Singapore was 7,965, 7,918, and 7,595 persons/km^2^, respectively, the population density of the U.S. was 35, 36, and 36 persons/km^2^, respectively, the population density of the U.K. was 276, 277, and 277 persons/km^2^, respectively (23). From 2019 to 2021, the proportion of population aged 65 and above to the total population in China was 12.02%, 12.60%, and 13.15%, respectively, that of Singapore was 12.20%, 13.15%, and 14.13%, respectively, that of the U.S. was 15.79%, 16.22%, and 16.68%, respectively, and that of the U.K. was 18.53%, 18.72% and 18.92%, respectively (23). Second, according to the Global Digital Economy Development Index Report (32), the U.S., Singapore, and the U.K. ranked the top three in terms of the overall index in 2021, and China ranked eighth. Meanwhile, in terms of the Digital Technology Index, China, Singapore, the U.S., and the U.K. ranked fifteenth, sixth, first, and eleventh, respectively. Third, from 2019 to 2021, the number of doctors per 1,000 people in China was 2.26, 2.39, and 2.52, respectively, that of Singapore was 2.5, 2.6, and 2.8, respectively, that of the U.S. was 2.64, 2.63, and 2.67, respectively, and that of the U.K. was 2.95, 3.03, and 3.18, respectively (24). From 2019 to 2021, the number of nurses per 1,000 people in China was 3.13, 3.3, and 3.52, respectively, that of Singapore was 7.5, 7.4, and 7.9, respectively, that of the U.S. was 11.97, 11.83, and 11.98, respectively, and that of the U.K. was 8.2, 8.46, and 8.68, respectively (24). From 2019 to 2021, the number of hospital beds per 1,000 people in China was 4.83, 5, and 5.2, respectively, that of Singapore was 2.45, 2.46, and 2.48, respectively, that of the U.S. was 2.8, 2.78, and 2.77, respectively, and that of the U.K. was 2.45, 2.43, and 2.42, respectively (24). In addition, the healthcare system in Singapore, the U.S., and the U.K. is highly structured and hierarchical (33–35). However, multilevel referrals in China are practically unrealized (36, 37). In the case of infectious disease outbreaks, the former can prevent the healthcare system from being overwhelmed and reduce the risk of virus transmission by avoiding non-essential in-person visits. Finally, China and Singapore are collective societies that strive for harmony and group belonging. The U.S. and the U.K. are individualistic societies that strive for freedom, independence, rights, and equality. Hence, compared to the U.S. and the U.K., cultural beliefs and values in China and Singapore make strict anti-epidemic measures an easier task.

Through the same procedure as in Section 3.1.1 for assessing the potential levels of resilience from scale, this paper derives the potential levels of resilience from structure for the four countries under various non-political factors and their combined effects, as shown in Table 3. According to Table 3, the potential levels of resilience from structure in China, Singapore, the U.S., and the U.K. are medium, high, high, and medium, respectively.

**Table 3:** Potential levels of resilience from structure.

**Country**	**Socio-demographic factors-derived resilience**	**Digital factors-derived resilience**	**Medical factors-derived resilience**	**Cultural factors-derived resilience**	**Potential level under combined effects**
China	Medium	Low	Low	High	Medium
Singapore	Low	Moderately high	Moderately high	High	High
The U.S.	High	High	High	Medium	High
The U.K.	Medium	Medium	Medium	Medium	Medium

The political factors are then analyzed to derive actual levels of resilience from structure. First, China adheres to a strict accountability system. Singapore has a well-established and strict accountability system. While the accountability systems in the U.S. and the U.K. are less stringent. Second, according to the 2019 Edelman Trust Barometer (38), the percentage of trust in government in China was 86%, that of Singapore was 67%, that of the U.S. was 40%, and that of the U.K. was 42%. Third, in China and Singapore, the government's ability to intervene in enterprises is very strong. But the same scenario is hardly likely to happen in the U.S. and the U.K. This is due to the fact that over the past two centuries, the entrepreneurial class has succeeded in taming the government, the “Leviathan,” into the “Night's Watch” by means of the constraints imposed on the executive power by party politics and legislation. Finally, government-society relations in China and Singapore are usually characterized by a “command-compliance” or “guidance-response” dynamic. While enabling efficient decision-making and execution, this model also faces challenges such as relatively constrained space for expressing diverse demands (39). In contrast, government-society relations in the U.S. and U.K. frequently involve dynamic negotiations of interests rather than merely compliance/response. While providing greater capacity to incorporate diverse interests, this model comes with associated costs such as prolonged decision-making processes and policy gridlock on certain issues (40).

Through the same procedure as in Section 3.1.1 for assessing the actual levels of resilience from scale, this paper derives the impact of key political factors on the actual levels of resilience from structure in the four countries during infectious disease outbreaks, as shown in Table 4. After considering the influence of political factors, we posit that the actual levels of resilience from structure in China and Singapore are higher than their potential levels, while the actual levels of resilience from structure in the U.S. and the U.K. are lower than their potential levels.

**Table 4:** Actual levels of resilience from structure.

**Country**	**Impact of administrative accountability system**	**Impact of political trust**	**Impact of government-market relations**	**Impact of government-society relations**	**Actual level of resilience**
China	In the event of infectious disease outbreaks, strict accountability systems in China and Singapore can facilitate better and stricter implementation of anti-epidemic measures by officials at all levels of government, although this can also lead to problems of the practice of formalities for formalities' sake and “one-size-fits-all.”	In the event of infectious disease outbreaks, compared with the U.S. and the U.K., the high degree of trust in government in China and Singapore is conducive to facilitating the strict and effective implementation of specific anti-epidemic measures.	In the event of infectious disease outbreaks, compared with Singapore, the U.S., and the U.K., China can not only quickly and indefinitely suspend non-essential production activities, but also open green channels to speed up the production of anti-epidemic materials.	In the event of infectious disease outbreaks, compared with China and Singapore, there is relatively more friction and conflict between government and society in the U.S. and the U.K., resulting in greater resistance to the implementation of anti-epidemic measures.	Higher than potential
Singapore					Higher than potential
The U.S.					Lower than potential
The U.K.					Lower than potential

**Figure 2:**
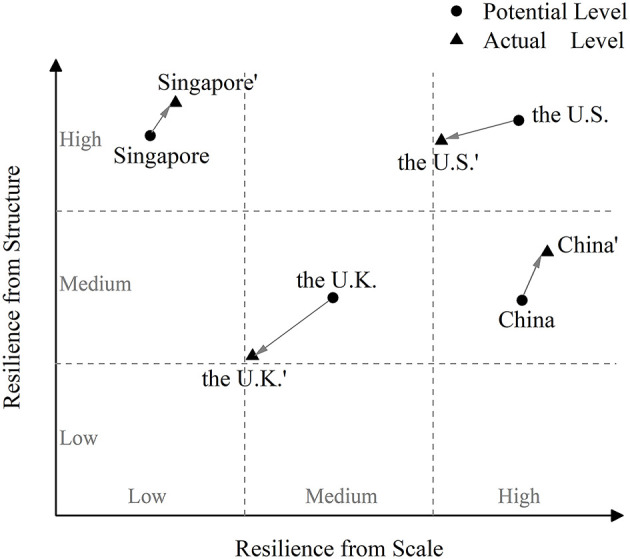
Levels of two types of resilience in four countries.

Accordingly, this paper draws a two-dimensional matrix diagram, as shown in Figure 2. This matrix diagram, with resilience from scale as the horizontal axis and resilience from structure as the vertical axis, contains only the first quadrant, i.e., the region where the two types of resilience show positive enhancement. Based on the above analytical results, this paper roughly marks the positions of the potential and actual levels of the two types of resilience in the four countries with dots on the diagram. It is important to note that countries at the same level do not mean that their resilience is identical. Similarly, countries with consistent directions of revisions in the actual level of resilience do not imply that the magnitude of their revisions is identical. The central purpose of this paper is to achieve relative comparability of the two types of resilience across the four countries, rather than to work toward a precise and unambiguous quantitative assessment of their levels. Therefore, the dots depicted in the diagram serve as visual indicators of the relative positions of the two types of resilience across the four countries, rather than conveying their precise absolute values.

### 3.2 Anti-epidemic policies in four countries from the perspective of two types of resilience

#### 3.2.1 Anti-epidemic policies in China

China's COVID-19 response encompassed three distinct stages, i.e., zeroing, dynamic zeroing, and co-existence with COVID-19. Each stage involved modifications to healthcare delivery and public health governance.

Stage 1 (from January 2020 to July 2021) centered on large-scale mobilization and centralized control, enabled by China's exceptionally high resilience from scale. On January 23, 2020, faced with the unknown and unprecedented surge of COVID-19, China closed outbound traffic from Wuhan City (hereafter Wuhan), i.e., lockdown. The lockdown was followed by other cities in Hubei Province (hereafter Hubei). Depending on its resilience from scale, China mobilized hundreds of medical teams, tens of thousands of medical personnel, and large quantities of medical supplies from various regions to assist Wuhan and Hubei. In order to halt the spread of the virus, China implemented travel restrictions and social distancing measures and pressed the “pause button” throughout society. In March 2020, the Chinese mainland reported no new domestically transmitted cases. From December 2019 to May 2020, there were 83,017 cumulative confirmed cases and 4,634 cumulative deaths in the Chinese Mainland, with an infection fatality rate of 5.6% (41). Since then, there were sporadic outbreaks in the Chinese Mainland due to imported cases abroad. While adhering to the “zeroing” policy, China adopted the response strategy of “preventing the coronavirus from entering the country and stemming its domestic resurgence.” China implemented closed-loop management measures in all aspects of air transportation and border crossings, insisting on the simultaneous prevention of people, objects, and the environment. Meanwhile, the zones involved in the outbreak were classified as high, medium, and low risk, matched with differentiated management measures and timely risk adjustment. In addition, China continued to improve its “health code” database by supplementing it with information on nucleic acid test results and travel experiences. On 31 December 2020, China granted conditional market approval to its first COVID-19 vaccine. Following this, China officially started vaccinations. This stage exemplified how resilience from scale supported aggressive suppression strategies in the face of uncertainty.

Stage 2 (from August 2021 to November 2022) focused on the “dynamic zeroing” strategy, driven by a significant enhancement in resilience from structure. This improvement was reflected in expanded testing capacity, faster and more accurate epidemiological investigations, increased isolation and treatment resources, widespread vaccination, and the growing operational proficiency of government officials and volunteers. Faced with more transmissible variants such as Delta and Omicron variants, China adopted a new policy called “dynamic zeroing” and mainly relied on its own resilience from structure to prevent and control COVID-19. Once an infected person was identified, China would conduct rapid and precise epidemiological investigations. And one or more rounds of nucleic acid tests would be carried out to identify all infected persons and cut off all transmission chains in communities. China accurately classified close contacts, secondary contacts, and general contacts, and delineated the scope of lockdown, control, and precaution zones to the smallest unit. Meanwhile, China continued to strengthen the research and development of antiviral drugs and vaccines and promote vaccination. From May 2020 to November 2022, there were 188,951 cumulative confirmed cases and 592 cumulative deaths in the Chinese Mainland (41). Excess deaths associated with COVID-19 between 1 January 2020 and 31 December 2021 were −52,063 (42). This stage represented a pivotal transition in strategy, moving away from broad, indiscriminate containment measures toward a precision-based approach characterized by data-driven interventions, localized responses, and adaptive risk management.

Stage 3 (from December 2022 to the present) signified a transition to sustainable co-existence with COVID-19, underpinned by the combined strengths of resilience from scale and structure. The Omicron subvariant BA.5 and later variants were termed “natural vaccines” in some scientific and media discourse. Its fatality rate was lower than that of seasonal influenza, while its infectivity was so high that it could not achieve “dynamic zeroing.” As of 11 November 2022, 90.26% of the population had been fully vaccinated in the Chinese Mainland. The level of nucleic acid tests, isolation, and treatment in China has been dramatically improved. Meanwhile, the world continued to be plagued by epidemics, with numerous cases imported from abroad. The livelihood issues caused by long-term, stringent, and even rigid anti-epidemic measures have gradually evolved into people's dissatisfaction with and doubts about the “dynamic zeroing” policy. Against this backdrop, China formally ended its “dynamic zeroing” policy and social control measures and officially entered the stage of “coexistence with COVID-19.” According to the Law of the People's Republic of China on Prevention and Treatment of Infectious Diseases, China downgraded the management of COVID-19 from top-level Class A to Class B. China no longer applied isolation measures to infected people and no longer determined close contacts or high-risk or low-risk zones. With the goal of “preserving health and reducing severe cases,” China further increased the vaccination rate, especially among the older adults. According to the Frontier Health and Quarantine Law of the People's Republic of China, China also removed COVID-19 from quarantinable infectious disease management. Since then, China has continued to carry out dynamic monitoring and early warning of COVID-19 outbreaks, keeping abreast of epidemiological trends, viral variants, and the supply of medical resources. To cope with new coronavirus strains and possible surges of infections, China issued a notice on the prevention and control of COVID-19 and other key infectious diseases in winter and spring in November 2023. Specifically, China has strengthened the implementation of epidemic prevention and control at ports of entry, continuous monitoring and early warning of epidemic dynamics, the prevention and control of key populations and key institutions, and preparations for medical treatment. This stage reflected a shift toward risk-based governance and long-term care redesign, supported by large-scale system capacity and adaptive delivery mechanisms, ensuring efficient resource utilization and public health continuity.

#### 3.2.2 Anti-epidemic policies in Singapore

Singapore's COVID-19 response progressed through three stages, i.e., containment, transition, and endemic management, involving policy recalibration, managerial coordination, and the integration of targeted measures into routine healthcare delivery.

Stage 1 (from January 2020 to May 2020) focused on rapid containment and centralized control, made possible by Singapore's high resilience from structure. In February 2020, Singapore raised Disease Outbreak Response System Condition (DORSCON) to Orange. Meanwhile, Singapore issued Stay-Home Notice (SHN) and progressively expanded its coverage. In March 2020, Singapore tightened borders and announced that all short-term visitors would now not be allowed to enter or transit through Singapore. In April 2020, Singapore implemented an elevated set of safe distancing measures, as a “Circuit Breaker” to curb the spread of the virus. The Circuit Breaker period lasted until May 4 and was then extended to June 1. In addition, Singapore promoted the research and development of antiviral drugs and vaccines. From January 2020 to May 2020, there were 34,884 cumulative confirmed cases and 23 cumulative deaths in Singapore (41). This stage illustrated how strong structural foundations facilitated effective policy-to-practice translation under high uncertainty.

Stage 2 (from June 2020 to February 2022) signified a transitional phase toward reopening, driven by the further enhancement of resilience from structure. Strengthened healthcare capacity, a well-advanced national vaccination programme, improved testing and contact tracing systems, and accumulating operational experience empowered Singapore to shift from rigid containment to a more flexible and risk-sensitive approach. In June 2020, Singapore exited the Circuit Breaker and implemented a cautious and calculated series of re-opening measures. Since then, “intermittent lockdown” was implemented through the cycling of initiation and cessation of social control measures. Specifically, in May 2021, as the number of locally transmitted COVID-19 cases and unlinked community cases continued to increase, Singapore went on Heightened Alert. In August 2021, Singapore undertook a mid-point review of the Heightened Alert measures and began to ease some of the measures in two steps. In September 2021, as daily cases started to rise exponentially from the end of August, Singapore entered the Stabilization Phase, which was extended from 25 October through 21 November 2021. In November 2021, Singapore further eased community Safe Management Measures (SMMs) and exited the Stabilization Phase into the Transition Phase. In January 2022, Singapore made a few further changes to vaccine policies and health protocols to further prepare to ride this Omicron wave. From June 2020 to February 2022, there were 689,540 cumulative confirmed cases and 996 cumulative deaths in Singapore (41). Excess deaths associated with COVID-19 between 1 January 2020 and 31 December 2021 were 1,475 (42). This stage exemplified delivery innovation, where the expansion of structural capabilities enabled agile, data-informed public health responses.

Stage 3 (from March 2022 to the present) marked Singapore's decisive transition to living with COVID-19, supported by resilience from scale and structure. Robust public health infrastructure, high vaccination coverage, and well-institutionalized response protocols enabled the government to systematically ease restrictions. On 24 March 2022, Singapore's Prime Minister Lee Hsien Loong made a speech and noted that the fight against COVID-19 reached a major turning point and Singapore would make a decisive move toward living with COVID-19. On 22 April 2022, the Singapore Ministry of Health (MOH) announced that the DORSCON level would be adjusted from Orange to Yellow. From 26 April 2022, Singapore stepped down almost all of SMMs. Singapore lifted vaccination-differentiated SMMs fully from 10 October 2022. In February 2023, around 80% of the population achieved minimum protection and around half were up to date with their vaccinations. Singapore adjusted the DORSCON level from Yellow to Green. Singapore also stepped down the remaining few COVID-19 measures and established an endemic COVID-19 new norm. At the end of 2023 and the beginning of 2024, COVID-19 infections continue to rise, with the highly contagious JN.1 coronavirus variant responsible for most cases. To preserve healthcare capacity, MOH has been working with public hospitals for contingency planning to maximize bed capacity for urgent cases in need of acute care. MOH has opened two COVID-19 Treatment Facility (CTF), the CTF at Singapore EXPO Hall 10 and the CTF at Crawfurd Hospital. Hospitals have tapped on step-down facilities and alternative care models to ensure proper right-siting of patients. Singapore also urges the public to exercise personal and social responsibility and keep their vaccination up to date to stay protected. This strategic shift illustrates care redesign grounded in long-term resilience, where structural efficiency and broad population compliance allowed for stable, large-scale adaptation to viral persistence.

#### 3.2.3 Anti-epidemic policies in the U.S.

The COVID-19 response in the U.S. reflected a fragmented but adaptive public health trajectory, shaped by shifting federal leadership and decentralized governance. It unfolded across four key stages, each characterized by policy adjustment and institutional coordination.

Stage 1 (from January 2020 to March 2020) centered on rapid containment through strong resilience from structure. On January 29, 2020, President Trump announced the formation of the President's Task Force on the Novel Coronavirus. The Secretary of Health and Human Services (HHS) declared a public health emergency on January 31, 2020, under section 319 of the Public Health Service Act (42 U.S.C. 247d), in response to COVID-19. On March 1, 2020, Trump proclaimed that the COVID-19 outbreak in the U.S. constituted a national emergency. On March 16, 2020, Trump announced new nationwide guidelines for stopping the spread of the novel coronavirus, calling on Americans to avoid gatherings of more than 10 people, avoid eating and drinking in bars, restaurants, and public food courts, avoid discretionary travel, etc. Meanwhile, most states issued stay-at-home or shelter-in-place orders. Furthermore, the U.S. spared no effort in advancing the research and development of antiviral drugs and vaccines. From January 2020 to March 2020, there were 160,020 cumulative confirmed cases and 2,953 cumulative deaths in the U.S. (41). While population-scale mobilization was limited, centralized mandates such as stay-at-home orders, travel restrictions, and early research investments underscored the role of structural preparedness in initiating containment under deep uncertainty.

Stage 2 (from April to December 2020) marked a premature shift toward reopening, driven by political considerations but still anchored in structural efforts. In April 2020, Trump issued new guidelines and recommended states and localities started to reopen their economies. As of April 28, 2020, there were 1,002,498 cumulative confirmed cases and 56,749 cumulative deaths of COVID-19 in the U.S. (41). In May 2020, the Trump administration launched Operation Warp Speed (OWS) to facilitate the development, manufacturing, and distribution of COVID-19 countermeasures at an unprecedented pace. Meanwhile, Trump resumed his re-election campaign and restarted his large-scale rallies in swing states. As COVID-19 cases spiked, some states began re-closing some bars and restaurants and delayed any further re-opening phases. However, these states reopened their economies after seeing a downward trend of coronavirus cases, and so on and so forth. In December 2020, the FDA approved the first coronavirus vaccine. The U.S. officially began vaccination on 14 December 2020. From April 2020 to December 2020, there were 18,856,281 cumulative confirmed cases and 329,298 cumulative deaths in the U.S. (41). At this stage, resilience from scale remained weak, with fragmented state-level policies, inconsistent public messaging, and limited testing undermining coordination. Despite rising cases, reopening progressed unevenly across states, reflecting low societal alignment and limited operational cohesion.

Stage 3 (from January 2021 to February 2022) saw resilience from structure further strengthened under the Biden administration, due to the improvement of nucleic acid detection capacity and efficiency, enhancement of the production and supply of critical materials and devices, major advances in research and development of therapeutics and vaccines, dissemination and practice of personal protection knowledge, etc. However, despite these advances, resilience from scale remained underdeveloped, with varying public compliance and persistent political divides limiting sustained impact. Thus, resilience from structure remained its main virtue in preventing and controlling COVID-19. President Joe Biden signed a flurry of executive actions in his first 100 days in office, primarily aimed at increasing domestic manufacturing of anti-epidemic materials and promoting vaccinations. In July 2021, the 7-day average of new cases in the U.S. dropped all the way down to 10,000, and the 7-day average of deaths fell below 200, which were both the lowest since March 2020. Nevertheless, the epidemic situation in the U.S. deteriorated rapidly in the second half of the year with the arrival of the Delta and Omicron variants. In September 2021, the Biden administration required all businesses with 100 or more employees to ensure that every worker was either vaccinated for COVID-19 or submitted to weekly testing for the coronavirus. Federal workers and contractors, 17 million healthcare workers at hospitals and other healthcare settings, and teachers and staff at the Head Start early education program and other federally funded educational settings also were required to be vaccinated. In December 2021, Biden announced new actions to protect Americans and help communities and hospitals battle the Omicron variant. From January 2021 to February 2022, there were 60,010,681 cumulative confirmed cases and 617,525 cumulative deaths in the U.S. (41). Excess deaths associated with COVID-19 between 1 January 2020 and 31 December 2021 were 932,458 (42). The resurgence of cases due to variants like Delta and Omicron exposed the constraints of structural efforts absent broader societal mobilization.

Stage 4 (from March 2022 to the present) signified a transition to endemic management, underpinned by enhanced resilience from scale and structure. In March 2022, the Biden administration released the National COVID-19 Preparedness Plan and officially moved toward coexistence with COVID-19. The Biden administration lifted bans and shutdowns and mainly focused on tests, treatments, vaccinations, and variant suppression. The U.S. continued to produce vaccines and testing kits quickly and safely. In May 2022, the cumulative number of deaths from pneumonia in the U.S. exceeded 1 million (41). In December 2022, the Biden administration announced COVID-?19 Winter Preparedness Plan. The COVID-19 public health emergency ended on May 11, 2023. The federal coronavirus response was restructured and the coronavirus was viewed as an endemic public health threat that could be managed through the normal authority of agencies after the end of the COVID-19 public health emergency. At the end of 2023 and the beginning of 2024, the U.S. once again experienced a major COVID-19 surge, and the JN.1 coronavirus variant is responsible for most cases. Data from the CDC also showed that COVID-19 viral activity in wastewater was at a very high level. Several states have reinstated the requirement to wear masks. The CDC calls on the public to take steps to protect themselves against JN.1 and other circulating variants, including getting an updated COVID-19 vaccine, testing for COVID-19, and increasing space and distancing. The Biden administration issued a national preparedness plan, lifted emergency declarations, and institutionalized routine prevention, treatment, and surveillance systems. With high vaccine availability, mature testing capacity, and increased public awareness, the U.S. moved toward decentralized, risk-based governance. Although variant-driven surges continued, the systemic foundation enabled stable adaptation.

#### 3.2.4 Anti-epidemic policies in the U.K.

The COVID-19 response in the U.K. demonstrated a reactive yet progressively adaptive public health trajectory, shaped by fragmented institutional coordination, evolving governance priorities, and dynamic public sentiment. Its response could be divided into four distinct stages, each reflecting shifts in policy orientation, operational capacity, and epidemic control strategy.

Stage 1 (from January to mid-March 2020) was characterized by limited action due to low resilience from both scale and structure. By slowing down the spread of the virus rather than containing it completely, the U.K. hoped to delay the peak of the new coronavirus outbreak and decrease as much as possible its huge strain on the healthcare systems, especially with the flu season approaching. The U.K. allowed schools to remain open and public gatherings to continue as usual. Public Health England (PHE) required individuals to self-isolate for 7 days if they returned with symptoms from areas known to have COVID-19 cases, or without symptoms if the area was categorized as high risk. The U.K. did not test patients with mild symptoms and all testing capacity would be “pivoted” to hospital patients. From January to mid-March 2020, there were 3,269 cumulative confirmed cases and 144 cumulative deaths in the U.K. (41). The lack of preparedness and limited institutional mobilization reflected fragile systemic capacity under initial viral shock.

Stage 2 (from mid-March to May 2020) saw a shift toward stringent interventions, primarily driven by rising resilience from scale. Not only did the British economy not grow as a result of the virtually non-existent anti-epidemic measures, but the spread of the virus led to varying degrees of serious damage to the local medical resources, the employment environment, and the price index, which triggered extreme discontent and anger among the British public. On March 23, 2020, Prime Minister Boris Johnson announced a 3-week lockdown in the U.K. People would be allowed to leave their homes for very limited purposes. Meanwhile, the U.K. increased stocks of testing kits and equipment and accelerated the search for treatments and vaccines. On April 16, 2020, the U.K. announced that the nationwide lockdown imposed to slow the spread of the new coronavirus would remain in place for at least three more weeks. On May 7, 2020, the nationwide lockdown continued for another 3 weeks. On May 28, 2020, the Department of Health and Social Care launched Test and Trace designed to ensure that anyone with symptoms of COVID-19 had access to a test. From mid-March to May 2020, there were 257,915 cumulative confirmed cases and 36,770 cumulative deaths in the U.K. (41). While institutional structures lagged, expanded resource mobilization enabled broader population-level interventions.

Stage 3 (from June 2020 to January 2022) marked the rise of resilience from scale and structure, due to the scientific understanding of the virus, the increase in medical personnel and hospital beds, the availability of testing and antivirals, prior anti-epidemic experience, the vaccination programme, the increased level of population immunity, etc. At this stage, the U.K. adopted a “pulsed” anti-epidemic strategy, i.e., imposing a lockdown when the epidemic situation was serious and then lifting the lockdown after a slight easing, and so on and so forth. The U.K. mainly relied on its own resilience from scale to prevent and control COVID-19 when imposing a lockdown. And the U.K. mainly relied on its own resilience from structure to prevent and control COVID-19 when lifting a lockdown. In June 2020, the U.K. began easing lockdown restrictions. In September 2020, the number of cases climbed significantly. The U.K. developed the “rule of six,” limiting all social gatherings, except for in a limited number of circumstances, to just six people. On October 31, 2020, the U.K. reached a million COVID-19 cases (41). On the same day, Boris Johnson announced that England would come under a second national lockdown lasting 4 weeks. As lockdown came to an end on December 2, 2020, the U.K. became the first country in the world to approve the use of a coronavirus vaccine. On December 8, 2020, the U.K. officially began vaccination. In January 2021, the Prime Minister announced a national lockdown again. Meanwhile, the U.K. accelerated the vaccination programme at pace. In March 2021, lockdown easing began. In July 2021, almost all remaining social control measures in the U.K. were lifted. In September 2021, the Prime Minister set out the government's plan to manage COVID-19 throughout autumn and winter, including plans A and B. Plan A was activated to steer the country through autumn and winter 2021-22. In December 2021, the Prime Minister announced a move to Plan B in England as Omicron spreads across the U.K. In January 2022, the Prime Minister announced that Plan B restrictions in England ended as Omicron was in retreat. From June 2020 to January 2022, there were 17,057,978 cumulative confirmed cases and 118,984 cumulative deaths in the U.K. (41). Excess deaths associated with COVID-19 between 1 January 2020 and 31 December 2021 were 148,897 (42). Regulatory flexibility, public health innovation, and adaptive risk governance defined this phase.

Stage 4 (from February 2022 to the present) reflected a shift toward institutional normalization, supported by established resilience from scale and structure. In February 2022, the Prime Minister published its strategy, living with COVID-19, and confirmed next steps for living with COVID-19. In England, all remaining domestic restrictions in law were removed. From April 1, 2022, free symptomatic and asymptomatic testing for the general public also ended, except for the oldest age groups and those most vulnerable to COVID-19. Scotland, Wales, and Northern Ireland subsequently lifted their social control measures. Since then, the U.K. has experienced several coronavirus variants and surges of infections. The U.K. has continued to protect the most vulnerable with targeted vaccines and treatments and track the virus in granular detail. Meanwhile, the U.K. managed and responded to future risks through more routine public health interventions, including enhancing border controls, strengthening the cleaning and disinfection of public places, and encouraging the public to keep their distance and wear masks in public places. Responses became increasingly standardized, grounded in existing health interventions and public adaptation to sustained viral presence.

## 4 Discussion and conclusions

As the most serious global infectious disease pandemic since the 1918 influenza pandemic, COVID-19 caused widespread illness and death, as well as economic, social, and political disruptions. On May 5, 2023, the WHO announced that COVID-19 no longer constituted a public health emergency of international concern. The vast majority of countries worldwide have ended the interventions that have been used to contain COVID-19 on a societal level and transitioned from emergency mode to managing COVID-19 alongside other infectious diseases. The world is returning to a life similar to that before the pandemic. However, the magnitude of the impact caused by COVID-19 has varied a lot between countries. Prior to coexisting with COVID-19, some countries have been very successful in limiting the spread of disease and preventing deaths, such as China and Singapore. Some scholars believe that this may serve as evidence that authoritarianism is more conducive to the prevention and control of infectious disease outbreaks than liberal democracy, despite its obvious disadvantages in terms of stimulating social vitality and promoting technological innovation (43). However, this study contends that such binary classifications oversimplify the complex institutional, organizational, and political dynamics underlying national responses. Rather than attributing outcomes to regime type alone, we develop and apply a dual-resilience framework-resilience from scale and resilience from structure-to explain the differential policy trajectories of China, Singapore, the U.S., and the U.K. Our findings suggest that the timing, intensity, and flexibility of COVID-19 responses were largely shaped by how these two types of resilience evolved in each country. As the risk of future pandemics remains high, these insights have practical implications. Building national resilience requires not only investment in public health infrastructure and surveillance, but also institutional mechanisms that enable agile, coordinated, and legitimate crisis response. The COVID-19 pandemic should therefore be seen not as a singular event, but as a stress test that revealed deeper systemic capacities-and vulnerabilities-that will shape responses to future global health crises.

The contributions and potential implications of this study are as follows. First, this paper advances a novel dual-dimensional resilience framework to better assess health system resilience and pandemic response, transcending reductive political regime typologies (e.g., democratic vs. authoritarian) and superficial outcome metrics (e.g., case/death counts). It reveals the dynamic change of health system resilience before and during crises, offering a more nuanced explanation for anti-epidemic policy variation than do static regime labels or lagging indicators. Second, this paper underscores the interdependence of scale and structure dimensions in shaping pandemic response. Comparative analysis of China, Singapore, the U.S., and the U.K. reveals that neither dimension operates in isolation, but their synergy determines outbreak response quality. The study demonstrates how deficiencies in one dimension undermine the other, explaining paradoxical outcomes, such as nations with immense scale potential struggling due to structural fragmentation, or structurally agile systems constrained by scale limitations. Third, this paper highlights the critical role of both political and non-political variables in explaining national capacities to respond to infectious disease threats. This research decisively moves beyond simplistic politicization of pandemic response by identifying and analyzing the complex interplay of both political and non-political variables shaping national capacities.

Admittedly, there are several limitations in this paper. First, the exclusive focus on four countries inherently limits the study's generalizability and external validity, particularly for low- and middle-income countries (LMICs). Future research should test and validate the proposed dual-dimensional resilience framework across diverse socioeconomic settings, particularly in LMICs. This requires explicitly incorporating resource and structural constraints and collaborating with researchers and practitioners in LMICs to co-design context-specific validation studies, thereby enhancing the framework's cross-contextual applicability and robustness. Second, the assessment of resilience levels via expert consultation introduces inherent subjectivity. While valuable for nuanced insights, this approach remains vulnerable to individual biases, differing interpretations of “scale” and “structure,” and difficulties standardizing evaluations across national contexts, potentially compromising comparative precision. Third, the analysis is anchored primarily on the COVID-19 pandemic response. While highly relevant, the findings regarding the two resilience dimensions and their interplay might be influenced by the specific characteristics of this singular event. The framework's validity and the generalizability of lessons learned require further testing against responses to different types of infectious disease outbreaks over varying timeframes to confirm its robustness across diverse threats.

## Data Availability

The original contributions presented in the study are included in the article/supplementary material, further inquiries can be directed to the corresponding author.
